# Use of Demineralized Autologous Dentin Graft with the Tooth Processing Device in Immediate Post-Extraction Implant Placement and Loading: A 12-Month Evaluation of Peri-Implant Soft Tissue Stability

**DOI:** 10.3390/ma19204310

**Published:** 2026-10-13

**Authors:** Giuseppe Bavetta, Stefano Di Carlo, Giulia Amelina, Francesca De Angelis, Luca Piccoli, Nicola Pranno, Enrico Guerrieri, Edoardo Brauner

**Affiliations:** 1 Private Practice, Studio Dentistico Associato Bavetta, 90143 Palermo, Italy; giuseppe@studiobavetta.com; 2 Department of Oral and Maxillofacial Sciences, Sapienza University of Rome, Via Caserta 6, 00161 Rome, Italy; stefano.dicarlo@uniroma1.it (S.D.C.); francesca.deangelis@uniroma1.it (F.D.A.); luca.piccoli@uniroma1.it (L.P.); nicola.pranno@uniroma1.it (N.P.); guerrieri.1679821@studenti.uniroma1.it (E.G.); edoardo.brauner@uniroma1.it (E.B.); 3 Implanto-Prosthetic Unit, Policlinico Umberto I, Viale Regina Elena I 287b, 00161 Rome, Italy

**Keywords:** immediate implant placement, autologous dentin graft, peri-implant soft tissue

## Abstract

**Purpose:** To evaluate the effectiveness of immediate implant placement and loading combined with autologous dentin grafting in reducing post-extraction tissue remodelling, with particular emphasis on peri-implant soft tissue stability in the esthetic zone. **Materials and Methods:** This retrospective observational study included 13 patients (14 implant sites) with Type 1 extraction sockets in the anterior maxilla. Following atraumatic tooth extraction, implants were immediately placed and grafted with autologous dentin processed from the extracted tooth using the Tooth Transformer device. Screw-retained provisional restorations were immediately delivered through a digital workflow, and definitive restorations replicated the provisional transmucosal profile. Soft tissue dimensional changes were evaluated by superimposing stereolithography (STL) files from intraoral scans acquired before treatment and after at least 12 months. Vertical changes were measured as a single value, while horizontal changes were assessed 2 and 4 mm apical to the gingival margin. **Results:** All implants remained clinically stable, with no biological or prosthetic complications. Mean vertical soft tissue collapse was 0.69 mm, while mean horizontal collapse was 0.71 mm at 2 mm and 0.57 mm at 4 mm apical to the gingival margin. In most cases, dimensional changes were below 1 mm, with greater contour alterations at the more coronal level. **Conclusions:** Within the limitations of this study, immediate implant placement and loading combined with autologous dentin grafting using the Tooth Transformer device resulted in limited peri-implant soft tissue alterations after 12 months. This protocol may represent a biologically favourable, minimally invasive approach for ridge preservation while avoiding donor site morbidity and heterologous grafting materials.

## 1. Introduction

It is well established that tooth extraction is followed by dimensional alterations of the alveolar ridge in both height and width, as the volume and morphology of the alveolar bone are closely related to the presence of the tooth. These biological changes have been extensively investigated in both animal models and human studies [1,2,3]. The extent of post-extraction remodeling varies considerably among individuals and may be influenced by local anatomical and biological conditions [4]. Clinically, these changes may compromise the available hard- and soft-tissue volume and, particularly in the anterior maxilla, may affect the functional and esthetic outcome of subsequent implant-supported rehabilitation.

Following tooth extraction, bone remodeling is characterized by an initial resorption of the bundle bone followed by new bone formation and progressive remodeling of the extraction socket. Schropp et al. reported that approximately two-thirds of the horizontal ridge reduction occurs within the first 3 months after extraction, with an overall reduction of nearly 50% after 12 months [5]. Most dimensional changes occur during the early healing period. Because the buccal cortical plate is generally thinner than the lingual or palatal plate and contains a high proportion of bundle bone, its resorption may result in both horizontal and vertical reduction in the alveolar ridge [1,2,3,4,5].

Post-extraction remodeling, however, is not limited to the hard tissues. Changes in the contour and dimensions of the overlying soft tissues may also occur during healing and are particularly relevant in the esthetic zone, where even limited alterations of the peri-implant mucosal profile may influence the final emergence profile and esthetic outcome [5]. Consequently, the management of extraction sockets should consider both the preservation of the alveolar ridge and the maintenance of the peri-implant soft-tissue architecture.

Several therapeutic strategies have therefore been proposed to limit the dimensional consequences of tooth extraction. These include preservation of the extraction socket and immediate or delayed implant placement, with the choice of treatment depending on the local anatomy, integrity of the socket walls, soft-tissue conditions, and prosthetic requirements. Surgical approaches for immediate implant placement include both flapless and flap elevation techniques, which have been investigated with regard to their effects on peri-implant hard- and soft-tissue outcomes [6]. Soft-tissue augmentation procedures, including connective tissue grafting and the use of soft-tissue substitutes, have also been proposed to improve peri-implant soft-tissue stability and esthetic outcomes following immediate implant placement [7]. Immediate implant placement may help reduce treatment time and facilitate prosthetically driven three-dimensional implant positioning when adequate anatomical conditions are present. In particular, immediate implant placement in sockets with an intact buccal bone wall has demonstrated predictable clinical outcomes. Under appropriate clinical conditions, immediate implant placement can also be combined with immediate loading. Evidence from systematic reviews indicates that immediate loading of single-implant restorations can achieve outcomes comparable to conventional loading protocols when adequate primary stability is obtained [8,9]. In the present protocol, immediate loading was performed when an insertion torque value of 20–45 Ncm and an ISQ > 60 were achieved [10,11,12].

The presence of a horizontal gap between the implant surface and the socket wall does not necessarily prevent osseointegration when appropriate anatomical conditions are present. Tarnow and Chu reported histological evidence of osseointegration in an immediate implant placed with an excessive gap distance without primary flap closure, graft, or membrane, provided that the buccal bone wall remained intact. Under these conditions, mesenchymal stem cells derived from the bone marrow and periosteal tissues can naturally colonize the extraction gap. To optimize this biological healing process and prevent post-extraction volumetric collapse, the circumferential gap between the implant surface and the socket walls in the present study was completely packed with autologous demineralized dentin graft. This graft provides an osteoconductive scaffold rich in bone morphogenetic proteins (BMPs) that facilitates MSC differentiation into osteoblasts, accelerating osseointegration while maintaining the facial bone architecture. This strategy aligns with the dual-zone therapeutic approach described by Chu et al., which emphasizes the simultaneous management of both the bone zone and the overlying soft-tissue zone. In this approach, a screw-retained provisional restoration functions as a socket-sealing device to support the peri-implant soft-tissue architecture while promoting stable physiological healing [8,9].

Grafting procedures may additionally be used to manage the extraction socket and the space between the implant surface and the socket wall. Several grafting materials have been investigated for alveolar ridge preservation and bone regeneration, including autologous, allogeneic, xenogeneic, and alloplastic materials. However, current evidence does not support a definitive consensus regarding the superiority of one grafting material over another, and the choice of graft should be considered in relation to the clinical indication and the characteristics of the defect [9,13]. Among these approaches, autologous tooth-derived grafts have gained increasing interest as an alternative source of grafting material. Autologous dentin can be obtained directly from the extracted tooth, allowing the tooth itself to be converted into a patient-specific graft without requiring a separate donor site [14]. Demineralized dentin matrices have been investigated as substrates for bone regeneration, and experimental evidence has supported their potential osteoinductive properties [15,16]. In particular, the Tooth Transformer device allows the extracted tooth to be processed into a demineralized dentin graft for use at the extraction site [17].

The treatment protocol investigated in the present study combined immediate implant placement, immediate loading, and grafting with autologous demineralized dentin processed from the extracted tooth using the Tooth Transformer device. The protocol also incorporated prosthetically driven guided implant placement and a screw-retained provisional restoration, with the aim of maintaining the peri-implant soft-tissue architecture during healing. The indications for immediate implant placement may also extend to extraction sockets presenting with chronic infection, provided that adequate debridement and decontamination are performed before implant insertion [17,18]. This further highlights the importance of careful socket evaluation and management of the local hard- and soft-tissue conditions when selecting an immediate implant protocol.

The aim of this retrospective observational study was to evaluate the peri-implant soft-tissue dimensional changes associated with this standardized protocol in Type 1 post-extraction sockets in the anterior maxilla. In particular, peri-implant soft-tissue dimensional changes were assessed by digital superimposition of STL files obtained before treatment and at a minimum follow-up of 12 months.

## 2. Materials and Methods

### 2.1. Study Design and Patient Selection

This retrospective observational study was conducted to evaluate peri-implant soft-tissue dimensional changes using digital STL models. A total of 13 patients (9 males and 4 females, aged 30–61 years) requiring immediate implant placement across 14 extraction sites (2 central incisors, 2 lateral incisors, 4 canines, and 6 premolars) were included in this study. All patients were non-smokers and presented without systemic comorbidities, including uncontrolled diabetes or bone metabolic disorders. Clinically, all treated sites exhibited a thick periodontal biotype with an intact buccal bone plate.

Patients presented with a compromised tooth in the anterior maxilla, between teeth 15 and 25, and a Type 1 post-extraction alveolar socket according to the Tarnow classification. Type 1 sockets were characterized by an intact buccal bone wall, preserved soft tissues, and the presence of the interproximal papilla. No quantitative measurement of buccal bone plate thickness was performed as part of the study protocol.

Tooth extraction was performed using a flapless and atraumatic technique, with the aim of preserving the integrity of the alveolar socket walls and surrounding soft tissues. Following extraction, the socket was carefully inspected to verify the integrity of the buccal bone wall and was subsequently debrided and curetted to remove residual tissue and debris.

Patients presenting with acute inflammatory conditions that contraindicated immediate implant placement were excluded. Cases with chronic periapical or periodontal infection were considered eligible provided that adequate debridement and decontamination could be performed before implant insertion.

All patients received antibiotic prophylaxis according to the protocol described by Casap [17]. Informed consent was obtained from all participants before inclusion in the study. Antibiotic therapy was initiated four days before surgery, with a daily dose of 1.5 g of amoxicillin or 0.9 g of clindamycin in patients allergic to penicillin and was continued for a total of 10 days.

The study protocol was approved by the Institutional Review Board of the Department of Dental and Maxillofacial Sciences (Department Council Resolution No. 84/2026; Protocol No. 0001265, dated 22 June 2026). The study was conducted in accordance with the principles of the Declaration of Helsinki.

This retrospective observational study was conducted and reported in compliance with the STROBE (Strengthening the Reporting of Observational Studies in Epidemiology) guidelines.

A limitation of the present study is that quantitative measurements of the buccal bone plate thickness and changes in peri-implant hard tissues were not performed. The study was specifically designed to evaluate peri-implant soft-tissue dimensional changes using digital STL superimposition. Although Cone Beam Computed Tomography (CBCT) was routinely performed for preoperative diagnostic and implant-planning purposes, postoperative CBCT examinations were not acquired solely for research purposes, as they were not clinically indicated and would have resulted in additional radiation exposure without therapeutic benefit. Consequently, changes in peri-implant bone dimensions could not be directly assessed. Nevertheless, the peri-implant soft-tissue contour is closely related to the underlying hard-tissue support, and changes in soft-tissue dimensions may therefore provide clinically relevant information regarding the stability of the peri-implant tissue complex, although they cannot be considered a substitute for direct assessment of the underlying bone.

### 2.2. Clinical Workflow

The standardized clinical workflow included the following operative steps:

- CBCT scan of the dental arches;
- Intraoral scanning;
- Diagnostic wax-up;
- Surgical guide design;
- Tooth extraction;
- Alveolar socket debridement;
- Implant placement;
- Grafting with autologous dentin processed using a tooth-processing device;
- Screw-retained provisional restoration;
- Screw-retained definitive restoration.

After tooth extraction, the alveolar socket was carefully debrided and curetted to remove infected tissue and debris and to optimize the conditions for graft incorporation and implant osseointegration.

**Figure 1:**
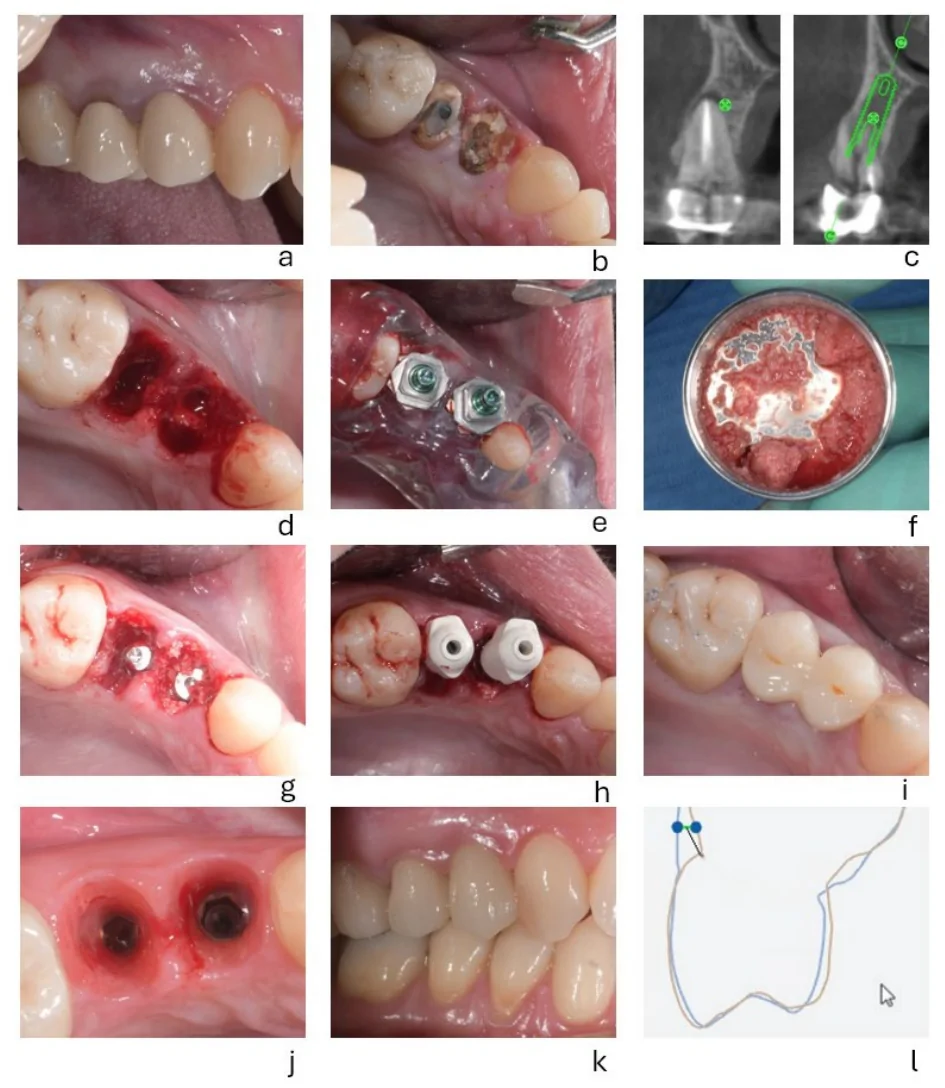
(**a**) Preoperative clinical situation. (**b**) Clinical situation after removal of the prosthetic bridge. (**c**) Digital implant planning at sites 1.4 and 1.5. (**d**) Post-extraction sockets. (**e**) Surgical guide in position with implants placed. (**f**) Autologous dentin grafting. (**g**) Implants placed and grafted with autologous dentin. (**h**) Scan abutments in position. (**i**) Screw-retained provisional restorations. (**j**) Three-month clinical healing. (**k**) Definitive restoration at 3 months. (**l**) Digital assessment of peri-implant soft tissue collapse at the 1-year follow-up, 2 mm apical to the gingival margin.

A representative clinical case illustrating the main steps of the treatment protocol is shown in Figure 1.

To ensure accurate implant positioning, a surgical guide was designed using Z3D/RealGUIDE software (version 5.0/6.0 3DIEMME S.r.l., Zimmer Biomet), which enables the registration of DICOM files obtained from the patient’s CBCT scan with STL files obtained from intraoral scans. A diagnostic wax-up corresponding to the provisional restoration was created within the software, followed by the design of a surgical guide allowing prosthetically driven implant placement. Implant diameter was selected according to anatomical considerations, with wider implants preferred whenever possible while maintaining a safety distance of 2 mm from the buccal bone wall and 1.5 mm from the adjacent teeth to preserve the peri-implant structures.

The implants used in this study were TSX implants (Zimmer Biomet, Warsaw, IN, USA), featuring a conical design and deep apical threads intended to provide high primary stability. Insertion torque values ranged between 20 and 45 Ncm, and all implants achieved an implant stability quotient (ISQ) > 60, allowing immediate loading according to the study protocol.

### 2.3. Autologous Dentin Grafting and Provisional Restoration

An autologous dentin graft was placed at the time of implant insertion. The extracted tooth was processed by grinding and demineralization using a tooth-processing device. According to the manufacturer’s protocol, a calibrated graft with a particle size of 0.4–0.8 mm (400–800 µm) was obtained. The average yield per processed tooth was approximately 1.5 g, the amount of graft material packed into each site was strictly adapted to completely fill the circumferential gap (jumping distance) between the implant surface and the socket walls without overpacking.

This material was selected because its autologous origin avoids the risk of immunological rejection and donor-derived disease transmission. Moreover, dentin naturally contains bone morphogenetic protein 2 (BMP-2) and type I collagen, which contribute to its osteoinductive and osteoconductive properties and may promote new bone formation. Recent studies have shown that the demineralization process does not compromise the osteoinductive potential of dentin [19]. Conversely, other studies have reported increased BMP-2 bioavailability following demineralization, potentially enhancing new bone formation [8].

Subsequently, a CAD/CAM-fabricated, screw-retained provisional restoration made of polymethyl methacrylate (PMMA) was positioned. The provisional restoration precisely replicated the morphology of the original tooth, including the root contour. The accuracy of the provisional restoration is critical, as it must effectively seal the post-extraction site and preserve the peri-implant soft tissue emergence profile [8]. For single-tooth cases, a prefabricated provisional restoration was delivered and screw-retained during the same surgical-prosthetic session. In cases involving two adjacent implants, an intraoral scan was taken after implant placement, and the provisional prosthetic restoration was delivered 4 h later. This approach was adopted to avoid potential interference during screw-retention or the need for intraoperative relining of the prefabricated provisional restoration.

### 2.4. Digital Workflow

After an appropriate healing period, six digital scans were acquired using a 3Shape intraoral scanner (3Shape A/S, Copenhagen, Denmark):

1. With the provisional restoration in place (PreOp1);
2. Without the provisional restoration (PreOp2);
3. With the match holder in place (Position scan);
4. Of the provisional restoration outside the oral cavity (Provisional scan);
5. Of the opposing arch;
6. In maximum intercuspidation.

The integration of these digital scans enabled the fabrication of the definitive restoration, accurately replicating the shape and dimensions of the transmucosal portion of the provisional restoration and thereby preserving the established emergence profile, as previously described [16]. STL files acquired before and after placement of the definitive screw-retained monolithic zirconia restoration were retrospectively compared to assess preservation of the peri-implant soft tissue profile.

## 3. Results

A total of 13 patients with 14 implant sites were included in the analysis. All sites were evaluated after a minimum follow-up of 12 months. No biological or prosthetic complications were recorded during the observation period. All implants remained clinically stable, and no cases of infection, graft loss, or soft tissue necrosis were observed.

**Figure 2:**
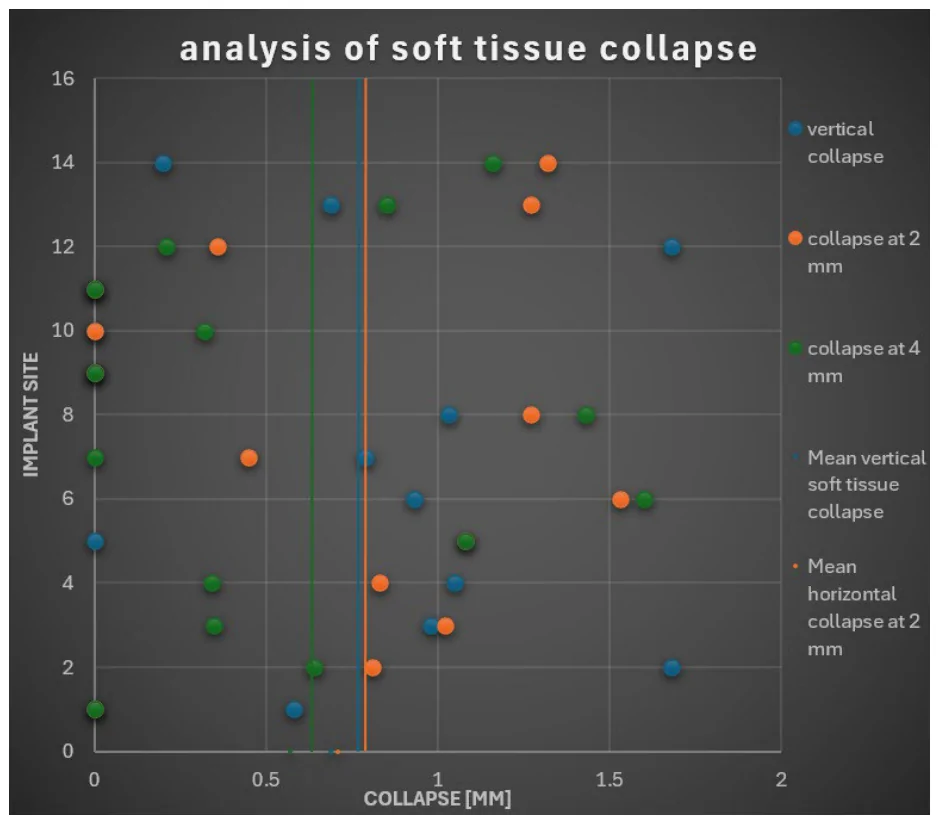
Individual peri-implant soft tissue collapse across all evaluated implant sites (*n* = 14). The horizontal axis indicates dimensional collapse in millimeters, while the vertical axis represents individual implant sites (numbered 1 to 14). Blue data points represent vertical soft tissue collapse, orange points indicate horizontal collapse at 2 mm, and green points denote horizontal collapse at 4 mm. Vertical reference lines represent the mean values for each parameter: blue line for mean vertical collapse, orange line for mean horizontal collapse at 2 mm, and green line for mean horizontal collapse at 4 mm.

Quantitative assessment of peri-implant soft tissue changes was performed by superimposing intraoral STL files acquired before treatment and at follow-up. Measurements focused on vertical soft tissue collapse and horizontal soft tissue collapse at 2 mm and 4 mm apical to the gingival margin. The individual measurements for all implant sites are reported in Table 1. The normality of the continuous variables was assessed using the Shapiro–Wilk test. Normally distributed data were expressed as mean ± standard deviation. As no significant departure from normality was detected, the data were expressed as mean ± standard deviation. To further illustrate the distribution of tissue alterations across all treated cases, the individual soft tissue dimensional changes relative to the overall sample means are visually depicted in Figure 2.

### 3.1. Vertical Soft Tissue Changes

The mean vertical soft tissue collapse was 0.69 mm, with values ranging from 0.00 to 1.68 mm. Most implant sites exhibited limited vertical soft tissue changes, with the majority showing variations of less than 1 mm.

### 3.2. Horizontal Soft Tissue Changes at 2 mm

At 2 mm apical to the gingival margin, the mean horizontal soft tissue collapse was 0.71 mm, with values ranging from 0.00 to 1.53 mm. Several implant sites showed no measurable horizontal variation at this level, whereas others exhibited a moderate reduction in soft tissue contour.

### 3.3. Horizontal Soft Tissue Changes at 4 mm

At 4 mm apical to the gingival margin, the mean horizontal soft tissue collapse was 0.57 mm, with values ranging from 0.00 to 1.60 mm. Horizontal soft tissue changes at this level were generally limited, with most implant sites showing variations of less than 1 mm.

## 4. Discussion

The present study investigated peri-implant soft tissue dimensional changes following immediate implant placement combined with autologous dentin grafting and immediate provisionalization. At a minimum follow-up of 12 months, the protocol was associated with limited vertical and horizontal soft tissue alterations, with dimensional changes generally remaining below 1 mm. The absence of a control group represents a limitation of the present study, as a comparative group would have allowed a clearer assessment of the specific effect of the treatment protocol. A randomized controlled design could also reduce potential selection bias and confounding factors; however, the retrospective nature of the present study did not allow for randomization or the inclusion of a predefined control group.

In two randomized clinical and volumetric trials by Fettouh et al. [19,20], peri-implant soft tissue changes following immediate implant placement in the esthetic zone were evaluated using intraoral scan superimposition at baseline and at 1-year follow-up. Both studies reported that immediate implant placement combined with prosthetically driven soft tissue support was associated with limited linear and volumetric soft tissue alterations and highlighted the role of peri-implant soft tissue volume in maintaining midfacial gingival margin stability. The 12-month evaluation window adopted in these trials and in the present study is widely recognized as a sufficient timeframe to capture the most significant physiological remodeling processes occurring post-extraction and to establish early soft tissue stability. The digital methodology and timing of evaluation adopted in these trials closely resemble those used in the present study, providing a useful basis for comparison of peri-implant soft tissue changes. However, while a 1-year follow-up is adequate for assessing short-term outcomes, the lack of longer-term observations remains a limitation, as extended follow-up is required to confirm the long-term persistence of these soft tissue contours against late recession or biological adaptation. Compared with these studies, the present investigation showed comparable magnitudes of linear soft tissue alteration, with mean horizontal reductions below 1 mm at both reference levels and mean vertical changes remaining below 1 mm at ≥12 months. Notably, a similar trend in the distribution of tissue changes was observed. In the present study, horizontal collapse was greater at 2 mm than at 4 mm apical to the gingival margin, indicating more pronounced dimensional changes in the coronal portion of the peri-implant soft tissues. This trend is consistent with findings from volumetric digital studies, in which greater contour changes were predominantly observed at more coronal reference levels. The superimposition of STL files obtained from intraoral scans has been reported as a reliable and reproducible method for assessing peri-implant soft tissue changes [17]. This digital approach allows the detection and quantification of limited horizontal and vertical contour variations over time and has been successfully applied in longitudinal clinical studies investigating peri-implant tissue dynamics in the esthetic zone [21].

### Comparison with Other Post-Extraction Approaches

Alveolar ridge preservation and gap filling during immediate implant placement can be achieved using various grafting materials. Autogenous bone is traditionally considered the gold standard due to its osteogenic, osteoinductive, and osteoconductive properties [13]; however, its clinical application is limited by donor site morbidity, harvested volume constraints, and unpredictable resorption. In this context, autologous demineralized dentin matrix processed via devices like the Tooth Transformer represents a biologically favorable alternative [15]. Human dentin shares a chemical composition strikingly similar to bone, consisting primarily of type I collagen and hydroxyapatite. The chemical demineralization process exposes trapped growth factors—specifically bone morphogenetic proteins (BMPs)—thereby conferring true osteoinductive capabilities while preserving osteoconductivity [18]. Furthermore, by recycling the patient’s own extracted tooth, this approach completely eliminates donor site morbidity and avoids the biological concerns associated with heterologous materials.

Most studies investigating post-extraction management strategies, including socket preservation, delayed implant placement, or spontaneous healing, primarily evaluate outcomes through radiographic assessment of hard tissue dimensional changes or clinical linear measurements of peri-implant soft tissues. Although these approaches provide valuable information on alveolar ridge remodeling, their outcome measures differ substantially from three-dimensional digital analyses of soft tissue contours based on intraoral scan superimposition. Therefore, quantitative comparisons between the results of the present study and those reported for conventional socket preservation techniques or spontaneous healing sites should be interpreted with caution due to these methodological differences. Nevertheless, the soft tissue stability observed in the present study generally compares favorably with outcomes reported for conventional approaches. The use of intraoral digital scanning provides a non-invasive approach for longitudinal assessment of peri-implant soft tissue contours without requiring additional radiographic examinations beyond those clinically indicated for patient care. This methodology may therefore offer a complementary means of evaluating soft tissue dimensional stability following immediate implant placement and autologous dentin grafting.

When interpreting the findings of the present study, several methodological limitations must be taken into account. First, the main limitation is its retrospective observational design and the absence of a control group, which prevents direct comparative conclusions regarding the clinical superiority of autologous dentin over other biomaterials. Second, the sample size was relatively small (13 patients, 14 implant sites), although sufficient to detect volumetric soft tissue trends. Third, blinding was not implemented during data collection and measurement analysis, even though potential operator bias was minimized by utilizing automated, objective computer-aided STL superimposition protocols. Finally, the inclusion of heterogeneous tooth sites across the maxillary arch (from position 15 to 25, including incisors, canines, and premolars) may introduce variability in local anatomy, flap tension, and tissue collapse dynamics.

## 5. Conclusions

Within the limitations of this retrospective observational study, the present protocol was associated with limited peri-implant soft tissue dimensional changes after a minimum follow-up of 12 months, while utilizing an autologous graft derived from the extracted tooth. This approach avoids donor site morbidity associated with autogenous bone harvesting and avoids the use of heterologous grafting materials [16]. Therefore, autologous demineralized dentin grafting may represent a biologically favorable and minimally invasive approach for ridge preservation in immediate implant placement and loading. Further prospective controlled studies with larger samples and longer follow-up periods are warranted to confirm these findings.

## Figures and Tables

**Table 1:** Peri-implant soft tissue dimensional changes at the 14 implant sites.

Implant Site	1	2	3	4	5	6	7	8	9	10	11	12	13	14	Mean + SD
**Vertical collapse (mm)**	0.58	1.68	0.98	1.05	0	0.93	0.79	1.03	0	0	0	1.68	0.69	0.2	0.69 ± 0.59 mm
**Horizontal collapse at 2 mm**	0	0.81	1.02	0.83	1.08	1.53	0.45	1.27	0	0	0	0.36	1.27	1.32	0.71 ± 0.56 mm
**Horizontal collapse at 4 mm**	0	0.64	0.35	0.34	1.08	1.6	0	1.43	0	0.32	0	0.21	0.85	1.16	0.57 ± 0.56 mm

## Data Availability

The original contributions presented in this study are included in the article. Further inquiries can be directed to the corresponding author.
